# Studies on the Detection of Oleuropein from Extra Virgin Olive Oils Using Enzymatic Biosensors

**DOI:** 10.3390/ijms232012569

**Published:** 2022-10-20

**Authors:** Alexandra Virginia Bounegru, Constantin Apetrei

**Affiliations:** Department of Chemistry, Physics and Environment, Faculty of Sciences and Environment, “Dunărea de Jos” University of Galaţi, 47 Domnească Street, 800008 Galaţi, Romania

**Keywords:** tyrosinase, laccase, screen-printed electrode, single-walled carbon nanotube, oleuropein, olive oil

## Abstract

Oleuropein (OLEU) is an important indicator of the quality and authenticity of extra virgin olive oils (EVOO). Electrochemical sensors and biosensors for the detection of oleuropein can be used to test the adulteration of extra virgin olive oils. The present study aimed at the qualitative and quantitative determination of oleuropein in commercial EVOO samples by applying electrochemical techniques, cyclic voltammetry (CV) and square wave voltammetry (SWV). The sensing devices used were two newly constructed enzyme biosensors, supported on single-layer carbon-nanotube-modified carbon screen-printed electrode (SPE/SWCNT) on whose surface tyrosinase (SPE/SWCNT/Tyr) and laccase (SPE/SWCNT/Lac) were immobilized, respectively. The active surfaces of the two biosensors were analyzed and characterized by different methods, cyclic voltammetry (CV), electrochemical impedance spectroscopy (EIS) and Fourier transform infrared spectroscopy (FTIR) and the results confirmed the efficient immobilization of the enzymes. SPE/SWCNT/Tyr was characterized by a low detection limit (LOD = 9.53 × 10^−8^ M) and a very good sensitivity (0.0718 μA·μM^−1^·cm^−2^) over a wide linearity range from 0.49 to 11.22 μM. The process occurring at the biosensor surface corresponds to kinetics (h = 0.90), and tyrosinase showed a high affinity towards OLEU. The tyrosinase-based biosensor was shown to have superior sensitive properties to the laccase-based one. Quantitative determination of OLEU in EVOOs was performed using SPE/SWCNT/Tyr and the results confirmed the presence of the compound in close amounts in the EVOOs analysed, proving that they have very good sensory properties.

## 1. Introduction

Carbon nanotubes (CNTs) have attracted the attention of researchers due to their unique physical and chemical properties. They are increasingly being used to build analytical devices for the detection of various biomolecules [1,2,3]. CNTs have a tubular structure composed of a graphene monomer deposited either in a single layer (SWCNT) or in the form of several layers (MWCNT) [4]. Having a larger diameter, CNTs provide a high surface-to-volume ratio and improve the signal-to-noise ratio compared to planar structures [5]. In addition, the modification of electrodes with SWCNTs has improved the performance of sensing, biocompatibility [6], structural flexibility [7] and axial electrical conductivity [8,9]. SWCNT-based biosensors generally have faster electron transfer kinetics than planar-structured carbon materials, higher sensitivity, lower detection limit [10] and lower cost [11,12].

Laccase and tyrosinase are enzymes often used to modify sensors functionalized with different types of carbon nanomaterials [13,14,15,16] and SWCNT [17,18,19]. These types of biosensors lend themselves very well to voltammetric methods and can contribute to the development of sensitive and specific devices for in situ analysis [15,20].

Both enzymes are polyphenoloxydases capable of catalyzing the oxidation of the o-diphenol group to o-quinone and are often selected for the electrochemical determination of phenolic compounds [21,22] in different types of samples, food [23,24,25], drugs [26,27,28], environmental samples [29,30,31], etc.

Olive oil (a product obtained from the fruit of the *Olea europeae* tree) is one of the most important elements in the Mediterranean diet, not only for its good taste and usefulness in gastronomy but also for its many beneficial properties due to its chemical composition [32,33]. In fact, the chemical composition of the oil and its sensory properties depend on the climate, but also on growing procedures, the variety of olive and production techniques [34]. EVOO has two major components: saponifiable and non-saponifiable compounds. The first group represents about 98% of the chemical composition of the oil and comprises triacylglycerols (TAG), mono- and di-glycerides, esters of fatty acids or free fatty acids and phosphatides, while the second group consists of hydrocarbons (squalene), phytosterols (e.g., β-sitosterol, stigmasterol and campesterol), tocopherols, pigments (chlorophylls and carotenoids), aliphatic and triterpene alcohols, triterpene acids (oleanolic acid), volatile compounds and polyphenol compounds. All of these compounds contribute, albeit in different ways, to the oil’s aroma and taste as well as its health benefits [35,36].

The hydrophilic phenolic fraction, which includes polyphenols, gives extra virgin olive oil beneficial effects on human health, such as antioxidant [37], anti-inflammatory [38,39], cardioprotective [40,41], hypolipidemic [42] antimicrobial [43,44] antineoplastic [45,46] or platelet antiaggregant effects. Phenolic compounds give the oil a bitter, pungent taste and a strong, fruity aroma, which represent special sensory qualities [47,48]. Increased content of polyphenols gives the oil high stability due to their antioxidant capacity and therefore a longer shelf-life [49].

The chemical structure and concentrations of polyphenols in EVOO vary according to several factors, including: fruit variety, geographical region, agricultural techniques, ripeness of olives at harvest and processing, and range from 0.02 to 600 mg/kg [50,51].

**Scheme 1:**
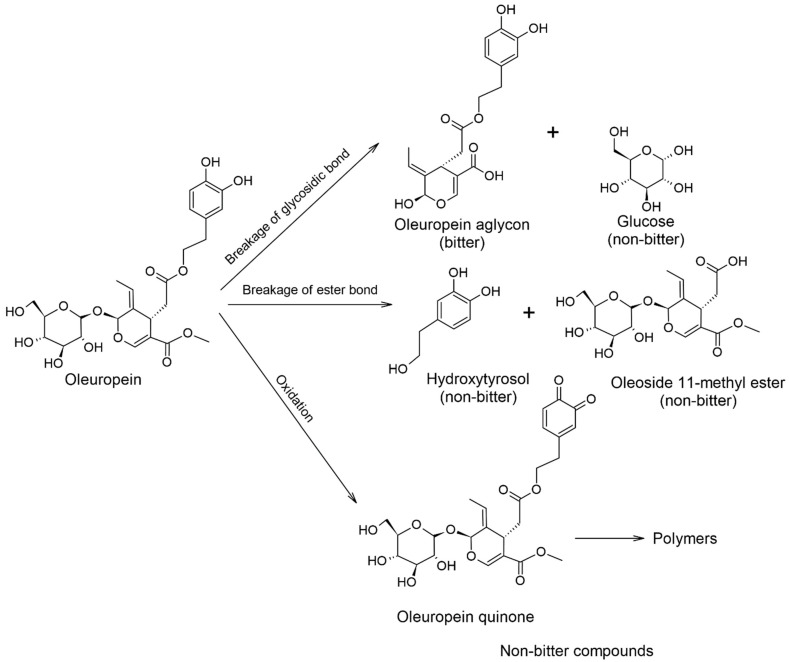
Bitterness of oleuropein and products of its chemical transformations. Addapted from [55,56].

Oleuropein (OLEU) is an important indicator for the level of ripeness of olive fruit and therefore for certifying the quality and authenticity of the product [52]. An increased concentration of OLEU is evidenced by the very bitter taste of fresh olives, which are more difficult to consume. They must be washed and fermented to make them suitable for consumption. Through fermentation, the conversion of oleuropein to hydroxytyrosol, the glycoside of elenolic acid (oleoside-11-methyl ester) and oleuropein aglycone takes place under hydrolytic cleavage of the ester and glycosidic bond (Scheme 1). The fermentation process can take place spontaneously or industrially, microbiologically with *Lactobacillus plantarum* [53]. The result is a microbiologically safe final product with improved sensory attributes and therefore a suitable concentration of oleuropein [54,55].

Therefore, the development of accurate quantification methods for OLEU has become an important objective for researchers to help ensure a food product that is qualitative, authentic and suitable for maintaining consumer health.

The most recent work follows the determination of oleuropein by increasingly advanced and performative strategies using chromatographic methods, (high-performance liquid chromatography (HPLC) [57], normal-phase ultra-high performance liquid chromatography with quadrupole time-of-flight mass spectrometry [58], high-performance liquid chromatography with diode array detection (HPLC-DAD) method [59]), spectroscopic (UV–vis absorption spectroscopy [60], nuclear resonance magnetic spectroscopy (NMR) [57,61], vibrational spectroscopy techniques coupled to chemometrics [62]) and electrochemical (cyclic voltammetry [63], adsorptive stripping square wave voltammetry [52], and differential pulse voltammetry [64]).

While chromatographic and spectroscopic methods involve complex and costly sample preparation steps that require longer working time, electrochemical methods have the advantage of using fewer reagents, are cheaper and provide results in a shorter time. Moreover, accurate experimental results have been obtained using electrochemical sensors that can be enhanced, modified or functionalized with different nanomaterials (carbon black [64], carbon nanotubes [52], metal nanoparticles [65]), polymers (chitosan [65]) or enzymes (tyrosinase [66], lipase [67]) to enhance electrocatalytic performance.

The development of enzyme biosensors based on carbon nanomaterials has important advantages that can be exploited for superior analytical performance. Specifically, the quinones formed as a result of oxidation reactions catalyzed by enzymes such as laccase or tyrosinase are electrochemically reduced to a low potential, and the measured current intensity is directly proportional to the concentration of the polyphenol in the sample [68], e.g., oleuropein. Moreover, reduced quinones on carbon nanoparticles generate o-diphenols, which are reoxidized by enzymes located near carbon nanoparticles, amplifying the redox process with lower detection limits [69].

To the best of our knowledge, no tyrosinase-based biosensors are reported in the literature for the quantification of oleuropein from real EVOO samples, so this paper aimed to develop a tyrosinase-based biosensor, supported by a screen-printed electrode previously modified with single-layer carbon nanotubes (SPE/SWCNT/Tyr). Moreover, the novelty of the work consists in comparing the results obtained by SPE/SWCNT/Tyr on the detection of oleuropein with those of another type of laccase-based enzyme biosensor (SPE/SWCNT/Lac), constructed in a similar manner. The electrocatalytic performance, sensitivity, selectivity and stability of the two newly constructed biosensors will be analysed, and finally, the oleuropein concentrations in real EVOO samples will be determined using the biosensor with superior properties and better feasibility.

## 2. Results and Discussion

### 2.1. Characterization of Enzymatic Biosensors

After the construction of the biosensors, characterization of the modified surfaces was performed by several methods. Cyclic voltammetry was the first method by means of which the evidence of enzyme immobilization on the supporting electrode was followed. Considering the previously published literature [68,70,71], it was decided to use the potential range from −0.4 to +1.3 V, proving to be optimal for the newly developed biosensors in this study. The signal was stable (after 3 cycles), with no interference or background noise, suggesting no contamination during the modification steps. Therefore, this potential range was also maintained in the study of oleuropein electrochemical reaction kinetics.

Regarding the optimal pH for electrochemical analysis, the literature reports that for tyrosinase a pH of 7.0 [68,72,73] prevents enzyme degradation and provides a strong and stable signal. Unlike tyrosinase, laccase maintains its optimal activity at pH 5.0 [71,74,75]. Therefore, all solutions were prepared using PBS 10^−1^ M at pH 7.0 and 5.0, respectively, depending on the working electrode used (SPE/SWCNT/Tyr, SPE/SWCNT/Lac).

**Figure 1:**
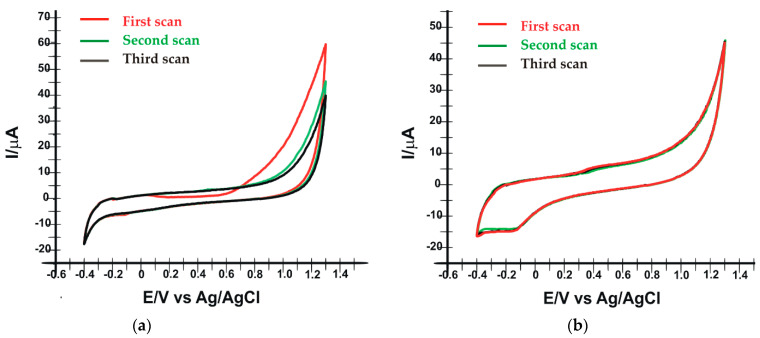
Cyclic voltammograms recorded in 10^−1^ M PBS solution, by the SPE/SWCNT/Lac at pH 5.0 (**a**) and SPE/SWCNT/Tyr at pH 7.0 (**b**). Scan rate 0.1 V·s^−1^.

In the case of laccase (Figure 1a), the oxidation peak appears only at the first scan at a potential *E*_pa_ = 0.914 V (*I*_pa_ = 13.913 μA), which is irreversible. At the second and third scans, oxidation and reduction peaks are no longer evident and the signal is stable.

Figure 1b shows the oxidation of tyrosinase at potential *E*_pa_ = 0.433 V (*I*_pa_ = 5.970 μA) and the reduction of the enzyme at *E*_pc_ = −0.171 V (*I*_pc_ = 14.739 μA). The redox process is quasi-reversible. In successive scans, no significant differences in the electrochemical behaviour of tyrosinase are observed.

Analyzing Figure 1, it can be stated that the enzymes were immobilized on the surface of the supporting electrodes, observing their electrochemical behaviour in both situations, but SPE/SWCNT/Tyr is characterized by a more stable signal and more obvious peaks.

In the next step, the two biosensors were analyzed by electrochemical impedance spectroscopy (EIS). EIS is one of the most important electrochemical techniques in which the impedance in a circuit is measured in ohms (as a unit of resistance). Compared to other electrochemical techniques, EIS is a steady-state technique, that uses a small signal and is able to record the signal over a very wide frequency range (e.g., 1 mHz–1 MHz). EIS can be used to explore charge transfer, mass transfer and diffusion processes. EIS, therefore, studies the intrinsic properties of nanomaterials or specific processes that could influence the conductance, resistance or capacitance of an electrochemical system. In a three-electrode system, EIS analysis is performed by fixing an applied voltage, and the Nyquist diagram obtained (used for the analysis of resistive systems), shows the resistance of the solution produced (*R*_s_), the charge transfer resistance (*R*_ct_) and the Warburg impedance (W) [76].

In this case, using two enzyme biosensors for detection, the redox process can be controlled predominantly by the diffusion of molecules, which can create an additional resistance known as Warburg impedance (W) (shown on the Nyquist diagram as a sloping line with a 45° slope) [76].

**Figure 2:**
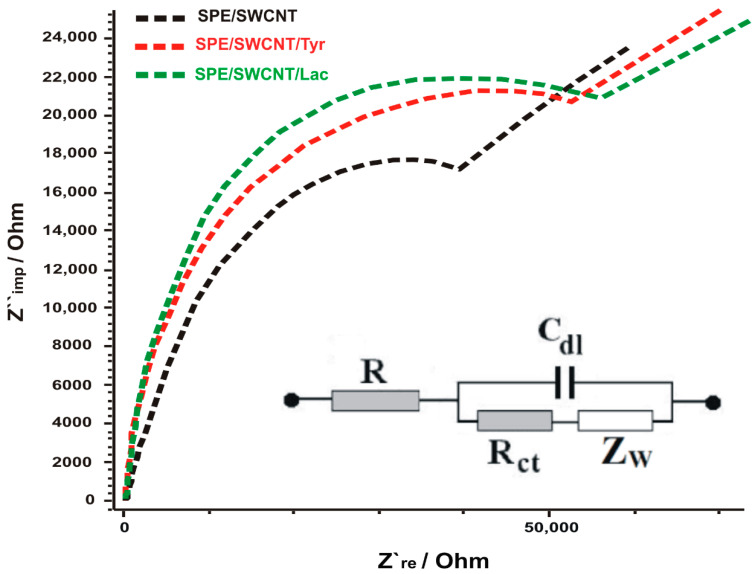
Nyquist plots of EIS for SPE/SWCNT (black line), SPE/SWCNT/Tyr (red line), SPE-SWCNT-Lac (green line) in 10^−1^ M KCl and 10^−3^ M [Fe(CN)_6_]^3−/4−^ for a frequency range of 0.01 Hz to 10 kHz, amplitude 10 mV. Inset: equivalent circuit applied to fit the impedance spectra.

The electrochemical processes occurring at the interface between SPE/SWCNT/Lac, respectively SPE/SWCNT/Tyr and the electrolyte chosen for analysis (in this case K_3_ [Fe(CN)_6_]/K_4_ [Fe(CN)_6_] 10^−3^ M and KCl 10^−1^ M in a 1:1 ratio) are transposed in the form of an equivalent circuit involving resistors, capacitors and inductor. The Randles equivalent circuit shows in a simplified way the solution resistance (*R*_s_), the double layer capacitance at the electrode surface (*C*_dI_), the charge transfer resistance (*R*_ct_) and the Warburg resistance (*Z*_w_), which is why it is most commonly used [77]. According to Figure 2, the Nyquist diagrams obtained in this study (in the form of a semicircle) correspond to a simulated Randles circuit, also shown in the figure.

The *R*_ct_ value was 39.615 Ω for the SPE/SWCNT electrode and for SPE-SWCNT-Tyr and SPE/SWCNT/Lac *R*_ct_ increases to 51,173 Ω and 54,200 Ω, respectively.

The higher values of *R*_ct_, still close in the case of biosensors, are attributed to the fact that enzymes are weaker electrical conductors at low frequencies, thus hindering electron transfer [78]. These results confirm the successful immobilization of enzymes on the surface of the supporting electrodes using glutaraldehyde [79]. This electrochemical behaviour of the biosensors is in agreement with other reported studies [78,80,81]. The difference in the value of *R*_ct_ between the two biosensors suggests that SPE-SWCNT-Tyr shows better electrocatalytic activity.

**Figure 3:**
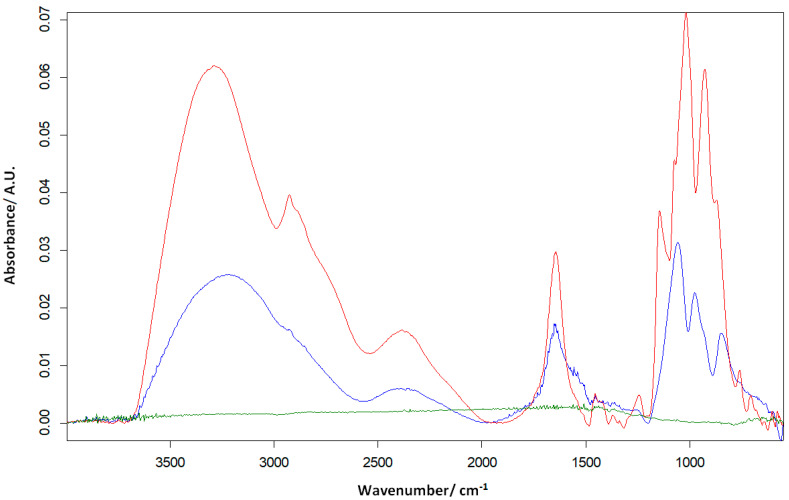
FTIR for SPE/SWCNT (green line), SPE/SWCNT/Tyr (blue line) and SPE/SWCNT/Lac (red line).

Another method that has been used to study enzyme immobilisation is the FTIR technique. SPE/SWCNT, SPE/SWCNT/Lac and SPE/SWCNT/Tyr (Figure 3) were analysed in turn, with the spectra recorded in attenuated total reflectance (ATR) mode in the range 4000–500 cm^−1^ with a resolution of 4 cm^−1^ and 32 scans.

The spectra of the two biosensors highlight the stretching vibrations of the C=N groups at about 1644 cm^−1^ (amide I, protein-specific band) [82], which indicates the interaction of glutaraldehyde with nitrogen atoms in the enzyme structures.

In the FTIR spectrum of SPE/SWCNT/Tyr, enzyme binding is also evident by the appearance of additional absorption bands at 1556 and 1539 cm^−1^ attributed to secondary amide bonds (C=N) [83] of tyrosinase with SWCNT via glutaraldehyde. The presence of these additional amide bands in SPE/SWCNT/Tyr confirms that the structure of tyrosinase is unchanged by the immobilization process and that the functionality of the enzyme is very little affected.

In addition, C-H stretching vibrations are observed in the FTIR spectra of both biosensors as a result of enzyme cross-linking, at wavelengths 3290 cm^−1^ (Lac) and 3215 cm^−1^ (Tyr), respectively. An obvious peak appears at 577 cm^−1^ expressing the Cu=N stretching vibration in the case of the laccase-based biosensor [84], which confirms the presence of laccase on the surface of the modified electrode. The bands appearing in the range 927–1144 cm^−1^, in the spectra of both biosensors, are assigned to C-O bond stretching vibrations.

### 2.2. Electrochemical Behavior of Oleuropein at SPE/SWCNT/Lac and SPE/SWCNT/Tyr

**Scheme 2:**
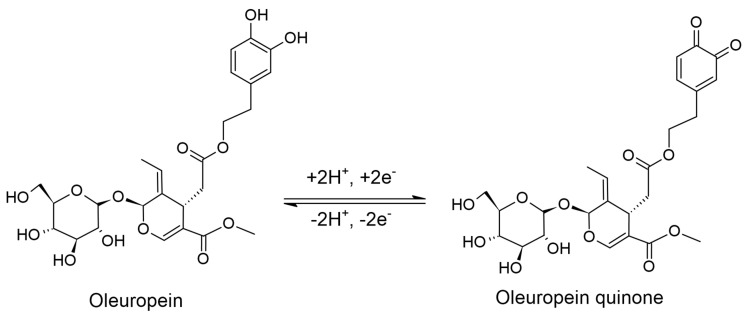
Reversible oxidation mechanism of oleuropein.

In this section, the electrochemical behaviour of oleuropein was studied using the two newly constructed biosensors, SPE/SWCNT/Lac and SPE/SWCNT/Tyr, by means of CV and SWV. For each electrode, a 10^−4^ M oleuropein stock solution was prepared at pH 5.0 (for Lac) and pH 7.0 (for Tyr). The mechanism of oleuropein oxidation in an aqueous solution is shown in Scheme 2.

**Figure 4:**
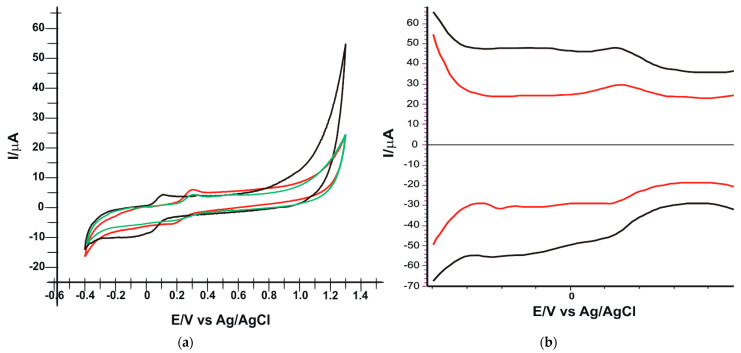
(**a**) Cyclic voltammograms of SPE/SWCNT (green line), SPE/SWCNT/Tyr (black line) and SPE/SWCNT-Lac (red line) in PBS solution 10^−1^ M (pH 7.0) containing 10^−4^ M OLEU. Scan rate: 0.1 V·s^−1^. (**b**) Square wave voltammograms of SPE/SWCNT/Tyr (black line) and SPE/SWCNT-Lac (red line) in PBS solution 10^−1^ M (pH 7.0) containing 10^−4^ M OLEU (anodic scan-up and cathodic scan-down).

CV was applied in the potential range from −0.4 to +1.3 V, at a scan rate of 0.1 V·s^-1^. Figure 4a shows the oleuropein oxido-reduction process for SPE/SWCNT (green line) SPE/SWCNT/Lac (black line) and for SPE/SWCNT/Tyr (red line) recorded by cyclic voltammetry. In all three cases, well-defined peaks corresponding to the oxidation and reduction of oleuropein are observed, with similar current intensities, but at noticeable different potentials.

Square wave voltammetry (SWV) was applied after prior optimization of the parameters: f (frequency) = 15 Hz; E_sw_ (applied pulse) = 90 mV.

Figure 4b shows the square wave voltammograms recorded with the same biosensors in 10^−4^ M oleuropein stock solution (PBS electrolyte 10^−1^ M).

The electrochemical behaviour of OLEU is confirmed by SWV. Peaks are reversible and well-defined with low background noise. The recorded voltammograms demonstrate that oleuropein can be identified and quantified by these voltammetric methods. The electrochemical parameters obtained with the two biosensors applying CV and SWV are presented in Table 1.

Analyzing the results obtained, it can be stated that the two enzymes have the ability to catalyze the redox process of OLEU, which is demonstrated by the value close to 1 of the ratio *I_pc_/I_pa_*, especially in the case of the laccase-based biosensor. Although the reversibility of the peaks is better in the case of SPE/SWCNT/Lac, both the oxidation and reduction peaks are well evidenced and occur at lower potentials in the case of SPE/SWCNT/Tyr, so the electron transfer occurring at the active surface is faster [68,85]. Until this stage of the study, it cannot be said with certainty that one of the two biosensors will have better electrochemical performance. The higher anodic peak and *I_pc_/I_pa_* ratio very close to 1 in the case of SPE/SWCNT/Lac could suggest a better sensitivity, but the lower potentials at which the peaks related to the oxidation–reduction process of oleuropein appear in the case of SPE/SWCNT/Tyr may suggest better selectivity for the analyte. Therefore, future experimental steps are essential to obtain clear comparative results of the two newly constructed biosensors.

Since the presence of immobilized enzymes predominantly influences the appearance and intensity of the cathodic current [70], which was also confirmed by SWV, where cathodic peaks were more evident, further analysis will be related to the cathodic current values.

### 2.3. Influence of Scanning Rates on Voltammetric Response

**Figure 5:**
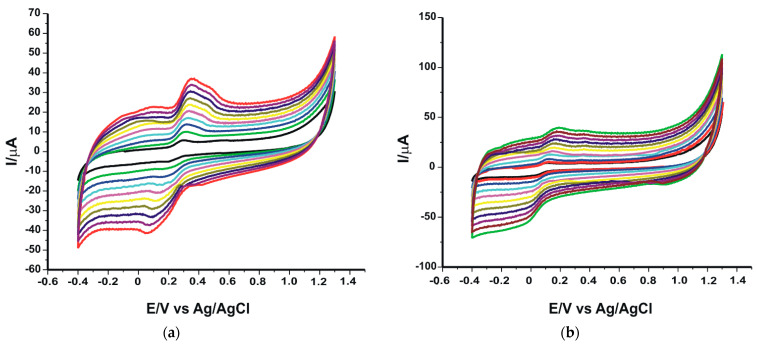
Cyclic voltammograms of SPE/SWCNT/Lac (**a**) and SPE/SWCNT/Tyr (**b**) in 10^−4^ M OLEU–10^−1^ M PBS solution. The cyclic voltammograms with different colours correspond to different scan rates.

An important influence on the electrochemical response provided by the newly developed biosensors is the applied scan rate. The study of kinetics can provide valuable information on the mechanism of the chemical reaction and the processes taking place at the active surface of the enzyme biosensor. For this step, the stock solution of OLEU 10^−4^ M, prepared under the same conditions as in the previous experiments, was used. The technique used was cyclic voltammetry, applying in turn several scanning rates in the range of 0.1–1.0 V·s^-1^. In each case, a progressive increase of the current intensity is observed with an increasing scanning rate (Figure 5).

**Figure 6:**
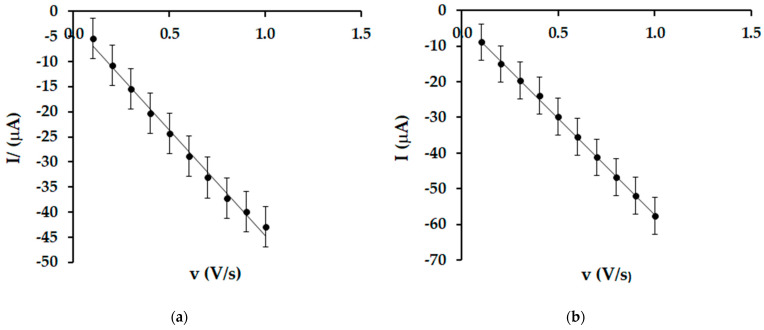
Linear dependence between cathodic current and scanning rate for SPE/SWCNT/Lac (**a**) and SPE/SWCNT/Tyr (**b**).

By analyzing the evolution of the cathodic currents, it was determined that there is a linear dependence between the cathodic currents and the scan rate (Figure 6) in both cases, suggesting that the process at the active surface of the electrospray is predominantly controlled by the oleuropein adsorption process. Several papers have reported this electrochemical behaviour of enzyme biosensors [86,87,88].

Subsequently, the surface coverage of the electrodes (Γ) with OLEU was calculated using Laviron’s equation [89]. The values of Γ are shown in Table 2.

It can be seen that the tyrosinase-based biosensor shows a higher OLEU adsorption coefficient, suggesting better electroanalytical properties. Both enzymes show selectivity for oleuropein detection, and SWCNT increases conductivity in both situations. The higher SPE/SWCNT/Tyr surface coverage can be explained by a better adhesion of tyrosinase to SWCNT or possibly a more efficient cross-linking, which translates into a better analyte–receptor affinity. However, the parameters measured and calculated so far demonstrate that both biosensors show great potential for sensitive and selective detection of oleuropein.

### 2.4. Calibration Curve and Detection Limit

To perform the calibration curve, square wave voltammograms of the two biosensors were recorded in oleuropein solutions in the concentration range of 0.01–28.62 μM.

For the quantification of oleuropein, square wave voltammetry was chosen because it implies a lower consumption of electroactive species, avoiding damage to the electrode surface. Furthermore, the oxidation or reduction peaks of oleuropein on the electrode surface are obtained in the same experiment, which increases the rate of the analysis [90].

For each biosensor, a stock solution of 10^−4^ M OLEU with the corresponding pH (5.0 for Lac and 7.0 for Tyr) was prepared from which different volumes were added to obtain solutions with different concentrations of OLEU.

**Figure 7:**
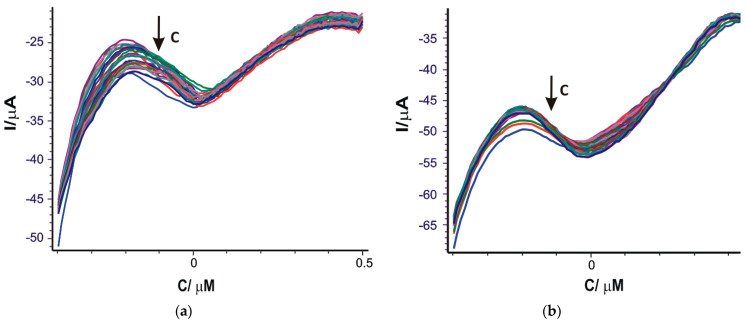
Square wave voltammograms recorded for SPE/SWCNT/Lac (**a**) and SPE/SWCNT/Tyr (**b**) on the concentration range 0.01–28.62 μM OLEU. Different colors correspond to different concentration of the analysed solutions.

As may be seen (Figure 7), the current of the cathodic peak increases with the increase in the OLEU concentration.

**Figure 8:**
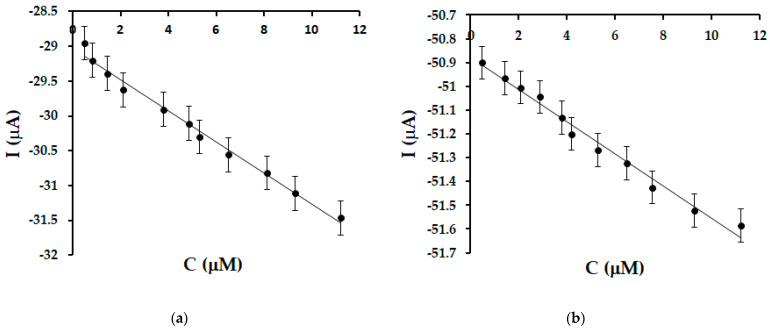
Linear fit in the range 0.49–11.22 μM for SPE/SWCNT/Lac (**a**) and SPE/SWCNT/Tyr (**b**).

In Figure 8 it can be seen that the cathodic peak current increases with increasing OLEU concentration. The concentration range was selected in accordance with the concentrations of oleuropein commonly reported in extra virgin olive oils.

A fairly wide linearity range was obtained in the range 0.49–11.22 μM with a coefficient of determination values greater than 0.96 for both biosensors.

We pursued the identification of a concentration range as close or identical as possible for the two biosensors and at the same time as wide as possible in which the linearity is satisfactory. The same linearity interval is important to ensure the same conditions for comparing the parameters that will be calculated (LOD and LOQ) for the two biosensors. Linear regression equations were used to calculate limits of detection (LOD = 3σ/m, where σ is the standard deviation and m the slope of the calibration curve) and limits of quantification (LOQ = 10 σ/m) [91] and are shown in Table 3.

SPE/SWCNT/Tyr is characterized by lower detection and quantification limits, confirming the very good electrocatalytic properties of this biosensor.

In the next step of the study, it was checked whether the general process at the surface of the biosensors corresponds to the Michaelis–Menten kinetics. For these determinations, data from the calibration curves of the two biosensors were used to calculate *I_max_*. Knowing the value of *I_max_*, log[*I*/(*I_max_* − *I*)] vs. log[OLEU] was plotted. From the equation of the line, the Hill coefficient (h) (identified as the slope of the line) was obtained.

Both SPE/SWCNT/Tyr and SPE/SWCNT/Lac show a Hill coefficient close to the value of 1, which confirms the kinetics at the biosensor level is Michaelis–Menten type. As shown in Table 4, SPE/SWCNT/Tyr shows an h less than 1, reflecting a negative cooperation effect between the active areas occupied by OLEU. SPE/SWCNT/Lac shows an h slightly greater than 1, reflecting a positive cooperation effect between the active centres of the biosensor surface. These values explain the affinity of the enzyme for oleuropein. Specifically, SPE/SWCNT/Tyr having an h < 1, shows a better selectivity, as tyrosinase once bound to oleuropein in solution, tends to have a lower affinity for other molecules possibly present in the sample.

Subsequently the Michaelis–Menten constant was calculated from the Lineweaver–Burk equation [73].

(1) $$\frac{1}{I}=\frac{1}{I_{max}}+\frac{K_{M}^{app}}{I_{max}\left[\mathrm{OLEU}\right]}$$

where: *I* is the cathodic current (A), *I_max_* is the steady-state current (A), $K_{M}^{app}$ is the apparent Michaelis–Menten constant and [OLEU] (M) is the substrate concentration.

Knowing the slope of the line obtained by plotting 1/[OLEU] vs. 1/I_pc_ and the value of *I*_max_ calculated previously, we obtained $K_{M}^{app}$ for each biosensor. $K_{M}^{app}$ is an affinity index between enzyme and substrate [92].

The measured and calculated parameters SPE/SWCNT/Tyr and SPE/SWCNT/Lac characteristics for OLEU are presented in Table 4.

Analyzing the results, it can be seen that in SPE/SWCNT/Tyr the concentration of tyrosinase that allows reaching a reaction rate equal to half of the maximum rate is much lower than in the case of immobilized laccase on SPE/SWCNT/Lac. Therefore, we can conclude that the tyrosinase on the SPE/SWCNT/Tyr surface shows a high affinity for oleuropein.

Considering that all the parameters calculated or measured with SPE/SWCNT/Tyr, showed better sensitivity and selectivity of this biosensor and a higher affinity of tyrosinase to the analyte, for quantitative determinations we will use this biosensor.

### 2.5. Stability and Precision of Studies

Stability is one of the major issues researchers face in the development of enzyme biosensors. Good stability increases the feasibility of the biosensor. In this case, the evaluation of the stability of the SPE/SWCNT/Tyr biosensor was performed in two ways. In the first case, the response to repeated immersions (20 successive measurements) in 10^−4^ M oleuropein solution was monitored. The cathodic current intensity corresponding to the presence of oleuropein decreased by 3.86%.

**Figure 9:**
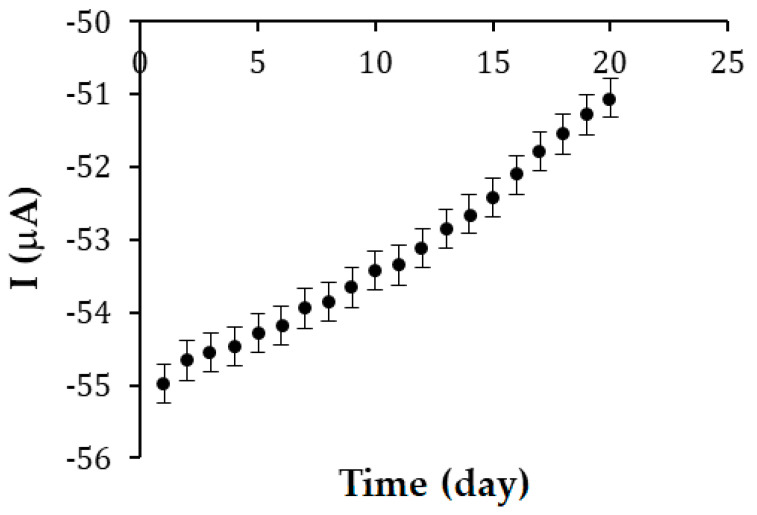
Cathodic current intensity obtained by immersing the same SPE/SWCNT/Tyr biosensor in a solution of 10^−4^ M OLEU, pH 7.0 over 20 consecutive days. The results are acceptable, considering that enzyme activity can easily degrade.

In terms of storage stability, the response of the same biosensor was evaluated for 20 days, with the analysis performed at approximately the same time. Between uses, the biosensor was stored at 4 °C. After 20 days, the biosensor response decreased by 7.12%. The recordings were made by square wave voltammetry. Figure 9 shows the evolution of the cathodic current intensity during the 20 days.

Concurrently, inter-day and intra-day precision studies were performed with SPE/SWCNT/Tyr in OLEU solution at the same concentration. Intra-day precision was evaluated by recording square wave voltammograms at 3-h intervals, and inter-day precision was evaluated for three consecutive days (first 3 days out of 20). The relative standard deviation (RSD %) value was less than 0.4%. Considering the first value obtained as the true value, the accuracy proved to be similar to the precision, also calculating an RSD with a value lower than 0.4%.

### 2.6. SPE/SWCNT/Tyr Biosensor Selectivity

To evaluate the selectivity of the biosensor for OLEU detection in real samples, the influence of two phenolic compounds (tyrosol, hydroxytyrosol and *p*-coumaric acid), present in EVOO, was studied. The OLEO concentration was 10^−4^ M and the detection technique was SWV. According to the literature, the potential corresponding to the reduction peak of hydroxytyrosol is approximately −0.003 V [70], that of thyrosol −0.200 V [93] and that of *p*-coumaric acid 0.011 V [71], which suggests that a low concentration of these compounds would not influence the biosensor response for OLEU. Different amounts of interferents were added, and the results confirm that up to a concentration of 10^−4^ M tyrosol,10^−4^ M hydroxythirosol and 10^−4^ M *p*-coumaric acid, the biosensor response is not substantially modified with an RSD of less than 3.8%. The results are presented in Table 5.

### 2.7. Determination of Oleuropein in EVOO

OLEU determination from real samples was performed by square wave voltammetry. The potential range was from 0.5 to −0.4 V, the applied pulse was 90 mV, the increment scan 7 mV and the frequency 15 Hz.

**Figure 10:**
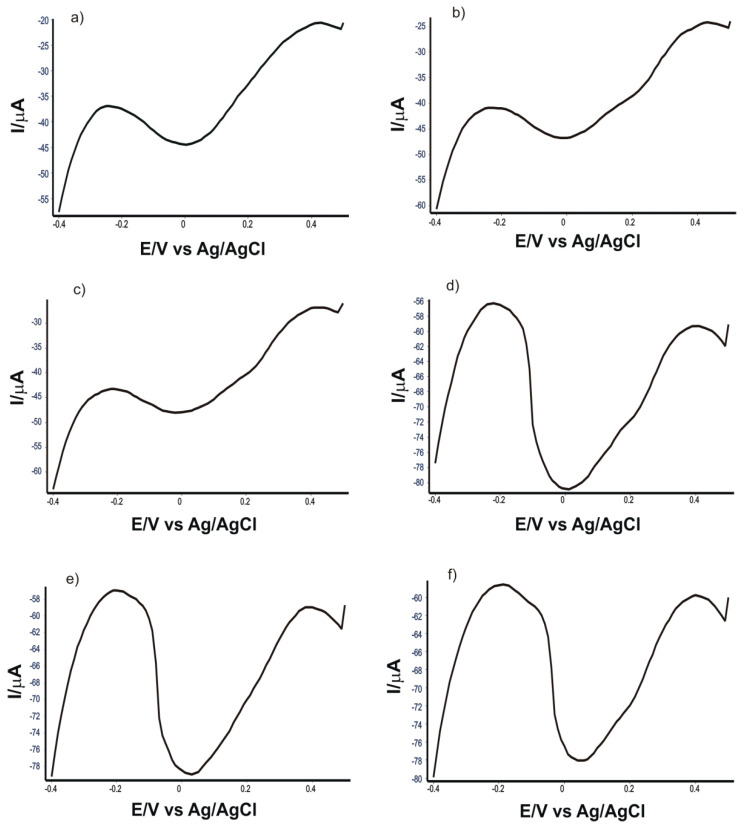
Square wave voltammograms of SPE-SWCNT-Ty immersed in solutions obtained from the 6 EVOOs selected for analysis: (**a**) Solaris, (**b**) Monini mini, (**c**) Costa D’oro Italiano, (**d**) Mueloliva, (**e**) Top Seller and (**f**) Regina Extra.

Figure 10 shows the square wave voltammograms of SPE-SWCNT-Ty immersed in solutions containing extracts obtained from the 6 EVOOs selected for analysis.

Using the cathodic current strengths at the potential corresponding to the presence of oleuropein and the slope of the right-hand side of the calibration equation, the amounts of oleuropein in the 6 types of EVOO were determined (Table 6).

Oleuropein is responsible for the pungent and slightly bitter taste of extra virgin olive oil. The amounts of OLEU determined in commercial EVOO are close, with the highest amount found in Regina Extra (Italy).

The precision expressed as relative standard deviation (RSD) ranged from 0.15 to 0.38%, indicating the repeatability of the method.

## 3. Materials and Methods

### 3.1. Reagents and Samples

NaH_2_ PO_4_ and Na_2_ HPO_4,_ reagents purchased from Sigma-Aldrich, St Louis, MO, USA, were used to prepare 10^−1^ M phosphate buffer solution (PBS), which was the support electrolyte in the electrochemical measurements performed.

The WTW pH meter (Weilheim, Germany) was used to adjust the pH to a value of 7 when the tyrosinase biosensor was used in the analysis and a value of 5.0 when the laccase biosensor was used. For EIS analysis, useful in the electrochemical characterization of modified electrodes, potassium chloride, potassium ferro- and ferricyanide purchased from Sigma-Aldrich, St Louis, MO, USA was used.

Oleuropein powder of analytical purity was purchased from Sigma-Aldrich, St Louis, MO, USA. A stock solution of oleuropein of concentration 10^−4^ M, prepared by completely dissolving the powder in 10^−1^ M PBS solution, was used for electrochemical analysis.

Laccase (from *Trametes versicolor*, 0.78 U/mg) and tyrosinase (T3824-25KU, from mushrooms, 8503 U/mg), lyophilized were purchased from Sigma Aldrich. For enzyme immobilization, a 50 μg/µL laccase solution (PBS 10^−1^ M solvent, pH 5.0) and an 80 μg/µL tyrosinase solution (PBS 10^−1^ M solvent, pH 7.0) were prepared in advance. The solutions were clear, and residue-free, with a yellowish-white colour in the case of the laccase solution, and a yellow–brown colour in the case of the tyrosinase solution.

Compounds of analytical purity, structurally similar to oleuropein (tyrosol, hydroxytyrosol and *p*-coumaric acid) were used for the interference studies, also purchased from Sigma-Aldrich.

### 3.2. Equipment

The newly developed biosensors were supported by screen-printed carbon-based electrodes (SPE/C) purchased from Metrohm DropSens (Oviedo, Spain). In the first step, SPE/C was modified with a suspension prepared from single-walled carbon nanotube powder purchased from Sigma-Aldrich (St Louis, MO, USA). The powder was dispersed in a mixture of dimethylformamide (DMF) (Sigma-Aldrich, St Louis, MO, USA) and ultrapure water (obtained with a Milli-Q system—Millipore, Bedford, MA, USA), yielding SPE/SWCNT. SPE/SWCNT was subsequently used to construct the two biosensors based on tyrosinase (Tyr) and laccase (Lac), respectively.

Electrochemical measurements were performed using an SP 150 potentiostat/galvanostat controlled by EC-Lab Express software with an electrochemical cell (50 mL) with three electrodes (reference electrode, auxiliary electrode and working electrode) connected.

Dissolving the substances and homogenizing the suspensions as efficiently as possible was carried out using an Elmasonic ultrasonic bath (Carl Roth GmbH, Karlsruhe, Germany).

A Bruker ALPHA-E FTIR spectrometer (BrukerOptik GmbH, Ettlingen, Germany) connected to OPUS software (BrukerOptik GmbH, Ettlingen, Germany) was used for the spectrometric characterization of the modified surfaces of the new biosensors. Spectra were recorded in attenuated total reflectance (ATR) mode in the range 4000–500 cm^−1^ with a resolution of 4 cm^−1^ and 32 scans. The ATR ZnSe crystal was cleaned with ultrapure water and isopropanol after each measurement.

### 3.3. Development of Enzymatic Biosensors

The first step to constructing enzyme biosensors was to modify screen-printed carbon-based electrodes (SPE/C) with single-layer carbon nanotubes. This procedure was achieved by dispersing on the active surface of the supporting electrode a 10 μL volume of a previously prepared suspension of SWCNT. The drop-and-dry process has been reported in several papers [70,94,95]. To prepare the SWCNT suspension, 10 mg of single-layer carbon nanotube powder was dispersed in a vehicle consisting of dimethylformamide and ultrapure water in a 1:1 ratio. For optimal dispersion, the suspension was sonicated for 30 min.

The established suspension volume (10 μL) was dripped onto the surface of the SPE/C electrodes using an Eppendorf micropipette in two successive steps. After each step, the electrodes were dried in a desiccator at 25 °C for 2 h.

**Figure 11:**
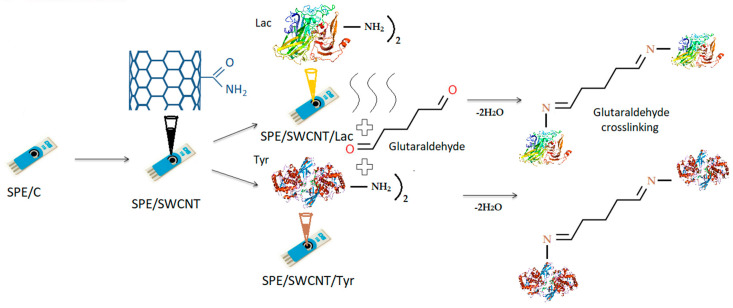
Preparation process of the tyrosinase and laccase biosensors based on a carbon-based screen-printed electrode.

The second step consisted of constructing two types of enzyme biosensors by modifying SPE/SWCNT with laccase and tyrosinase, respectively. The procedure was similar to the first step. Enzyme solutions were added to the SWCNT/SPE surface by the casting technique, followed by cross-linking using glutaraldehyde vapour. After 5 μL of added enzyme solution, the electrodes were kept in the exicator for one hour for drying. Another 5 μL was added in the same way. Exposure to glutaraldehyde vapour was for 1 min for each electrode. Cross-linking ensures a favourable electrical connection of SWCNT with the enzyme. However, a longer exposure time could decrease enzyme activity due to changes in the three-dimensional structure of the heteroprotein [15,21]. After immobilization, the biosensors were stored at 4 °C until use, a maximum of 72 h [96]. Figure 11 shows the steps of the preparation process of the laccase and tyrosinase-based biosensors and the schemes of the reactions taking place between glutaraldehyde and enzymes at the active surface of the electrodes.

### 3.4. Methods of Analysis

Cyclic voltammetry (CV) was used to characterize the surfaces of the modified electrodes and to study the electrochemical behaviour of oleuropein. For CV, the optimized potential range was in the range of −0.4 and +1.3 V and for the study of kinetics, the scan rates varied between 0.1 and 1.0 V·s^-1^ increasing at each scan by 0.1 V·s^-1^.

Characterisation of the active surface of the new biosensors was also performed using Fourier transform infrared spectroscopy (FTIR) and electrochemical impedance spectroscopy (EIS).

The FTIR method consists of collecting the infrared spectrum of a sample by passing a beam of infrared light through the sample. Evaluation of the transmitted light reveals how much energy is absorbed at each wavenumber. The Fourier transform instrument can generate a transmission or absorbance spectrum that indicates at what IR wavenumbers the sample absorbs. The analysis of these absorption properties reveals details about the molecular structure of the sample but can also be used for quantitative determinations [97].

Electrochemical impedance spectroscopy (EIS) is a technique used for the analysis of interface properties related to biorecognition events occurring at the electrode surface, such as substrate–enzyme interaction, being very useful in the present study for the characterization of SPE/SWCNT/Lac and SPE/SWCNT/Tyr [76].

Calibration curve and detection studies of oleuropein in model solution and in real EVOO samples involved the application of square wave voltammetry (SWV). The choice of this method implies a high speed of analysis, lower consumption of analyte and better conservation of the electrode active surface. Moreover, a single SWV scan shows the degree of reversibility of the electron transfer reaction [71,98].

### 3.5. Preparation of real samples

Samples of virgin olive oils required prior preparation for analysis, specifically a liquid–liquid extraction [99]. This sample preparation step consisted of adding 2.5 g of each oil to a 40:10, *v*/*v*, methanol–water mixture (5 mL). The samples were centrifuged and the supernatant was recovered using a pipette and added to the electrochemical cell in 50 mL PBS 10^−1^ M at pH 7.0. Methanol was purchased from Merck (Darmstadt, Germany).

## 4. Conclusions

The quantification of oleuropein in extra virgin olive oil can be of real help for both the authenticity and quality monitoring of this food product, with it being a compound related to the organoleptic properties and beneficial effects of EVOO.

In this study, the SPE/SWCNT/Tyr biosensor showed superior results in all steps of the research, starting with the characterization of the modified electrode, electron transfer resistance, the affinity of the enzyme to the analyte and ending with better sensitivity and lower detection limit, which is why it was used for the quantification of oleuropein in commercial EVOOs. Characterization methods of the newly constructed biosensors confirmed the presence and activity of enzymes on the sensor surface. Both CV and SWV proved to be suitable, efficient and easy to apply for the determination of this phenolic compound. Commercial EVOOs showed close amounts of OLEU, but the Italian product Regina Extra stood out with the highest concentration value.

Therefore, the construction of these new enzymatic biosensors based on laccase and tyrosinase, respectively, and the reporting of the results, can be a starting point towards new perspectives in the rapid and efficient in situ quantification of oleuropein in olive oils at different stages of production.

## Figures and Tables

**Table 1:** The values of the parameters obtained from the cyclic voltammograms and square wave voltammograms of the two biosensors immersed in 10^−4^ M OLEU solution (the electrolyte support was 10^−1^ M PBS of pH 5.0 (SPE/SWCNT/Lac) and pH 7.0 (SPE/SWCNT/Tyr)).

Method	Electrode	*E_pa_* (V)	*E_pc_* (V)	*E_1/2_* (V)	*I_pa_* (µA)	*I_pc_* (µA)	*I_pc_/I_pa_*
CV	SPE/SWCNT/Lac	0.303	0.175	0.239	6.09	−5.415	0.88
	SPE/SWCNT/Tyr	0.105	−0.010	0.0685	4.437	−8.882	2.00
SWV	SPE/SWCNT/Lac	0.130	−0.269	0.199	29.481	−29.047	0.98
	SPE/SWCNT/Tyr	0.140	−0.299	0.224	47.380	−54.943	1.15

E_1/2_ = (E_pa_ + E_pc_)/2.

**Table 2:** Linear dependence equation between I and v, R^2^ and the two biosensors.

Biosensor	Linear Equation	R^2^	*Γ* (mol × cm^−2^)
SPE/SWCNT/Lac	*I* = −4.200 × 10^−5^ *v* −2.714 × 10^−6^	0.9934	8.150 × 10^−11^
SPE/SWCNT/Tyr	*I* = −5.394 × 10^−5^ *v* −3.318 × 10^−6^	0.9989	1.046 × 10^−10^

**Table 3:** Linear dependence equation, R^2^, LOD and LOQ for the two modified electrodes.

Biosensor	Sensitivity	R^2^	LOD (M)	LOQ (M)
SPE/SWCNT/Lac	0.2455 μA·μM^−1^·cm^−2^	0.9607	1.01 × 10^−7^	3.35 × 10^−7^
SPE/SWCNT/Tyr	0.0718 μA·μM^−1^·cm^−2^	0.9778	9.53 × 10^−8^	3.18 × 10^−7^

**Table 4:** Characteristic parameters of SPE/SWCNT/Tyr and SPE/SWCNT/Lac for OLEU.

Biosensor	h (Hill Coefficient)	Imax/μA	$K_{M}^{app}$/μM
SPE/SWCNT/Lac	1.21	−30.487	0.0213
SPE/SWCNT/Tyr	0.90	−51.282	0.0000307

**Table 5:** OLEU results in the presence of interfering compounds.

Interfering Compound	Ratio	Recovery (%)	RSD (%)	Ratio	Recovery (%)	RSD (%)
Hydroxytyrosol	1:0.5	96.28	2.68	1:1	94.87	3.71
Tyrosol	1:0.5	96.32	2.65	1:1	95.36	3.35
*p*-coumaric acid	1:0.5	97.86	1.52	1:1	96.59	2.45

**Table 6:** Concentrations of OLEU (n = 3) in commercial EVOO obtained by the voltammetric method.

Commercial EVOO	mg/kg OLEU Obtained by SWV	RSD (±%)
Solaris (Greece)	59.5	0.36
Monini mini (Greece)	65.0	0.21
Costa D’oro Italiano (Italy)	69.5	0.38
Regina Extra (Italy)	95.5	0.15
Top Seller (Spain)	92.8	0.24
Mueloliva (Spain)	90.0	0.22

## Data Availability

Not applicable.
